# Enrichment of Animal Diets with Essential Oils—A Great Perspective on Improving Animal Performance and Quality Characteristics of the Derived Products

**DOI:** 10.3390/medicines4020035

**Published:** 2017-06-02

**Authors:** Panagiotis E. Simitzis

**Affiliations:** Department of Animal Breeding and Husbandry, Faculty of Animal Science and Aquaculture, Agricultural University of Athens, 75 Iera Odos, Athens 11855, Greece; pansimitzis@aua.gr; Tel.: +30-21-0529-4427; Fax: +30-21-0529-4442

**Keywords:** essential oils, growth performance, animal products, microbial spoilage, oxidation

## Abstract

Food industry operates in a competitive market and is continually facing challenges to retain or even increase its market share. Consistent high-quality animal products are required to maintain consumer confidence and consumption. Enrichment of foods with bioactive compounds such as the essential oils appears to improve quality characteristics of the derived products and protects consumers against oxidation and bacterial spoilage effects. Synthetic additives are nowadays questioned due to their suspected carcinogenic potential, and therefore extensive research has been undertaken to identify safe and efficient alternatives. Aromatic plants and their respective essential oils belong to natural products and are generally used in pig, poultry, rabbit and ruminant nutrition. The inclusion of essential oils in livestock diets is nowadays becoming a common practice, since dietary supplementation has been proven a simple and convenient strategy to effectively inhibit the oxidative reactions or microbial spoilage at their localized sites. A wide range of essential oils contain bioactive compounds that have the potential to act as multifunctional feed supplements for animals including effects on growth performance, digestive system, pathogenic bacterial growth and lipid oxidation. However, further studies are needed to clarify their exact action and establish their regular use in animal production.

## 1. Introduction

The growing public concern over the potential health risks and environmental impacts caused by the excessive use of antibiotics as growth promoters in animal production has led to a ban of their dietary application within the European Union since 2006 with the intention to minimize the transmission and the proliferation of resistant bacteria via the food chain. However, industries involved in the animal production chain, including the primary producers, processors, distributors, and retailers are constantly searching for efficacious, safe and cost effective substances with similar properties. As a result, natural feed supplements derived from plants such as the essential oils (EOs) have been examined as alternatives in animal production for improving growing performance parameters and the quality characteristics of the derived products (meat, milk and eggs) [1].

EOs are quite complex mixtures constituted by several components, hence resistance is less likely to become a problem compared to a single synthetic compound. They mainly consist of terpenoids and a variety of low molecular weight aliphatic hydrocarbons (phenols, aromatic aldehydes, etc.) [2]. EOs have the potential of a possible therapeutic exploitation in a variety of conditions in animal production: they enhance production of digestive secretions, stimulate blood circulation, exert antioxidant properties, reduce levels of pathogenic bacteria counts, mitigate the levels of fermentation products (ammonia and biogenic amines), enhance precaecal nutrient digestion, improve the intestinal availability of essential nutrients for absorption and relieve animal from immune defense stress (less activity of the gut-associated lymphatic system) [3,4,5]. However, the mechanisms underlying these functions have not yet been thoroughly elucidated.

Factors such as geographical location, harvesting period, plant part used (seeds, leaf, root or bark) and method of isolation (cold expression, steam distillation, extraction with non-aqueous solvents, etc.) modify the chemical composition of the essential oil derived from the respective plant and may introduce substantial compositional differences between preparations from the same plant species. Source of variability is also the type and origin of the EO, the level of EO that is included in the animal diet, the composition and the digestibility of the basal diet, the level of feed intake and hygiene and environmental conditions [4,6].

## 2. Classification, Structure and Metabolism of EOs

Essential oils are volatile secondary metabolite fractions mainly extracted by steam distillation (but also cold pressing, fermentation and enfleurage) that have been extensively used in the cosmetic but also in the food industry. The most important active compounds of EOs are categorized into two chemical groups: (mono- and sesqui-)terpenoids and phenylpropanoids that originate from different precursors of the primary metabolism and are further synthesized through separate metabolic pathways. Terpenoids (limonene, thymol, carvacrol, linalool, etc.) are characterized as deriving from an isoprene unit, namely a basic structure of five carbons (C_5_H_8_) through the mevalonate pathway [7]. On the other hand, phenylpropanoids (cinnamaldehyde, eugenol, anethole, etc.) derive mainly from the phenylalanine that is synthetized by the shikimate metabolic pathway and are compounds with a chain of three carbons bound to an aromatic ring of six carbons [8].

## 3. Antimicrobial Effects of EOs

Control of animal products spoilage and pathogenic bacteria has been mainly achieved by the use of synthetic chemical agents in previous decades. The applications of these compounds are nowadays limited due to questions regarding their potent undesirable aspects such as carcinogenicity, acute toxicity, teratogenicity and slow degradation periods. Public awareness has therefore generated interest in the use of more naturally occurring compounds with a broad spectrum of antimicrobial properties that improve the quality characteristics and shelf life of animal products [9]. Essential oils belong to the category of natural antimicrobials and have already been extensively studied for their antimicrobial activities in food systems [2,10,11]. Specific circumstances in food systems such as lipid content, proteins, water activity, pH and enzymes can potentially diminish the efficacy of EOs [10].

EOs and their components are hydrophobic, a characteristic that enables them to partition lipids in the bacterial cell wall and mitochondria, leading to their accumulation in the lipidic layer and a disruption of the membrane integrity and ion transport processes, and resulting in disturbances of the cell osmotic pressure. In detail, a rapid dissipation of H+ and K+ ion gradients (proton motive sources) and depletion of the intracellular ATP pool is observed through the reduction of ATP synthesis and the increased hydrolysis. As a result, the trans-membrane electric potential in bacterial cell is reduced and the proton permeability of the membrane is increased slowing down bacterial growth. When the bacterial tolerance threshold is passed, the extensive loss of cell contents or critical molecules and ions leads to cell death [10]. Moreover, the existence of the hydroxyl group attached to a phenyl ring and its ability to release its proton are considered as crucial factors in disrupting normal ion transport across the cytoplasmic membrane and in inactivating microbial enzymes [10,12].

The above mechanisms of action are more effective against gram-positive bacteria because the hydrophobic compounds of EOs can directly interact with the cell membrane. On the other hand, the external cell wall of gram-negative bacteria is hydrophillic and as a result the hydrophobic (or lipophillic) compounds of EOs cannot easily penetrate into the bacterial membrane [10,13]. However, low molecular weight molecules of EOs can cross the bacterial cell wall by diffusion through membrane proteins or through the layer of lipopolysaccharides resulting in the disruption of membrane integrity [2].

Post mortem application of EOs in food systems contributes to the extension of shelf life by limiting the spoilage of foodborne pathogens [11]. However, during the last decade, dietary supplementation with EOs is constantly gaining ground since it is a simple and convenient way of introducing natural antimicrobials that enter the circulatory system, and are further distributed and retained in tissues. For example, dietary supplementation with the EOs derived from Oregano sp. could be used for improving the microbial hygiene of turkey breast fillets [14] or that of whole carcasses by reducing the microbial load of total viable bacteria and of specific pathogens as shown in broilers [15] and rabbits [16].

### 3.1. Effects of EOs on the Digestive System and the Gut Microbiota of Swine and Poultry

The effects of EOs dietary supplementation on gut microflora, morphology, enzyme activity and growth performance parameters have been already extensively examined (Table 1). In general, EOs appear to suppress harmful microorganisms, stimulate beneficial microbes such as *Lactobacillus* spp., regulate the activity of enzymes and protect gut villi, however without inducing significant positive effects on body weight gain. On the other hand, feed conversion ratio is generally improved. Lactobacilli are a group of bacteria that have long been known for their ability to activate the intestinal immune system and increase the resistance to diseases, in part through the release of low-molecular weight peptides which induce immune activation [17]. At the same time, the increased Lactobacilli counts contribute to the colonization resistance against pathogenic microbes by modifying the receptors used by them [18,19]. As shown by the literature, EOs display antimicrobial action against *Escherichia coli* [20], *Clostridium perfringens* [21] and *Eimeria tenella* [22] and prevent their adhesion, colonization and proliferation in the gut of broilers. The decreased numbers of pathogenic bacteria in the gut and the maintenance of a proper bacterial balance between the number of beneficial and harmful bacteria in the intestine appear to improve the ability of epithelial cells to regenerate villus and thus enhance intestinal absorptive capacity [23].

The gut is a pivotal organ system that mediates nutrient uptake and use by the animals. As presented in Table 1, essential oils beneficially affect the ecosystem of gastrointestinal microflora by controlling potential pathogens, alleviating the oxidative stress caused by them and stabilizing gut microbiota. Improved intestinal health further enhances availability of essential nutrients for absorption (increased villus length and gut surface) and animal growth performance parameters could therefore be improved [3,5,24]. Moreover, digestive secretions (saliva, bile, mucus, etc.) and enzyme (trypsin, amylase, lipase, etc.) activity are enhanced partially through the irritation of the epithelial tissues and the reduction in the depth of the crypts in the ileum, resulting in an increased gastric retention time of the ingested feed and a better nutrient absorption [25,26].

The exact reasons for the discrepancies shown in Table 1 are speculative but they could be attributed to different type and levels of essential oil, the variability in the concentration of the major bioactive compounds among the different parts (barks, leaves, flowers, etc.) of the plant used, the period of supplementation and the animal species. Moreover, the variability in the efficacy of essential oil on performance of farm animals could also be a result, among others, of the basal diet composition, feed intake level, hygienic standards and environmental conditions [4].

### 3.2. Effects of EOs on Rumen Fermentation

Ruminal microbial activity plays an important role in the synthesis of high-quality protein for ruminants. However, microbial fermentation could lead to significant energy and protein losses as methane and ammonia that are significant environmental pollutants. Essential oils could be used to manipulate ruminal metabolism and selectively inhibit rumen methanogenesis due to their antimicrobial properties. Methane (CH_4_) is a potent greenhouse gas with a global warming potential 21 times that of carbon dioxide (CO_2_) [93]. At the same time, enteric methane losses represent 2–12% of gross energy intake in ruminants depending on diet composition and feed intake [94]. It can be therefore concluded that the reduction of CH_4_ emissions through the application of EOs is beneficial both for the animals (improved feed efficiency and productivity) and for the environment (mitigation of greenhouse effects) [95]. Essential oils could also positively influence protein metabolism and reduce rumen ammonia levels and lead to a more efficient utilization of dietary nitrogen by inhibiting deamination, i.e., the breakdown of amino acids to NH_3_, possibly through the selective limitation of the activity of a specific group of bacteria within the rumen, the “hyper-ammonia-producing bacteria” (*Prevotella* spp., *Ruminobacter amylophilus*, etc.) at the level of attachment and colonization [96,97].

Several EOs (thyme, oregano, cinnamon, garlic, etc.) have already been used with the intention to reduce CH_4_ production. At low doses, ruminal fermentation is not affected by essential oils, whereas at high doses these compounds inhibit the target microbial species as well as most rumen microbes [98]. As it has been demonstrated in in vitro studies, although the effects of EOs are diet (forage:concentrate ratio), pH (more intense action at low pH values) and time (adaptation period) dependent, mitigation of methane emissions occurs at high doses (>300 mg/L of culture fluid) and is frequently associated with a decrease in total volatile fatty acids concentration and feed digestion. As a result, although EOs in high doses could exert positive in vitro effects on rumen fermentation, these doses result in negative implications on feed palatability, digestion and animal productivity when applied in vivo. At the same time, the levels of EOs that have elicited favorable fermentation responses in vitro are far too high for in vivo application due to their possible toxic effects and high cost [99]. As shown in Table 2, no effects on feed intake, average daily gain and total volatile fatty acids concentrations and rates (i.e., acetate to propionate ratio) are generally observed after the dietary supplementation of ruminants with EOs or their main components as single compounds or mixtures. Since no significant differences in feed intake and rumen characteristics are found, it can be concluded that generally no effects on rumen methanogenesis are induced. A possible explanation is the ability of rumen microbes to adapt and degrade EOs components [95].

## 4. Antioxidant Effects of EOs

Oxidation of lipids and free radicals’ production are natural processes that destroy the membrane structure, disturb transport processes and cause losses in the function of the cell organelles. Lipids and especially phospholipids present in cell membranes are particularly susceptible to oxidative damage that is positively correlated with the degree of unsaturation of the fatty acids. Polyunsaturated fatty acids (PUFAs) are responsible for the maintenance of physiologically important cell membrane properties including fluidity and permeability. The peroxy radicals react with PUFAs and form hydroperoxides (ROOH), which later decompose to produce the volatile non-radical aromatic compounds (aldehydes, alkanes, conjugated dienes, etc.) that adversely affect lipids, pigments, proteins, carbohydrates vitamins and the overall quality of animal products by causing loss of nutritive value and limiting shelf-life [124].

Although synthetic antioxidants (butylated hydroxyanisole (BHA), butylated hydroxytoluene (BHT), *tert*-butylhydroquinone (TBHQ), gallates, etc.) are traditionally used with the intention to delay, retard or prevent the negative effects of lipid peroxidation by scavenging chain-carrying peroxyl radicals or diminishing the formation of initiating lipid radicals, the demand for natural antioxidants has recently been increased. During the last decades interest in employing antioxidants from natural sources to increase the shelf life of foods is considerably enhanced due to consumer preference for natural occurring ingredients and concerns about the possible toxic effects of synthetic antioxidants.

Animal diet plays a crucial role in the inhibition of free radicals production in the organism and the derived products. The nutritional prevention of oxidizing stress and its implications suggests the optimization of the antioxidant levels in the feed. As illustrated in Table 3, quality of animal products is generally improved after the incorporation of oregano or rosemary EO into the diets of animals. EOs are rich sources of natural antioxidants, such as the phenolic compounds and due their high redox properties and chemical structure have the ability to neutralize free radicals, chelate transitional metals and quench singlet and triplet oxygen by delocalization or decomposition of peroxides [125,126]. As a result, they affect lipid metabolism in animal tissues by exerting beneficial effects on the antioxidative enzymes activity (superoxide dismutase, catalase and glutathione peroxidase) and by preventing the production of reactive oxygen species and off-flavors deriving from the oxidation of polyunsaturated fatty acids [127]. However, depending on type and dosage, EOs can act as prooxidants by disrupting the integrity of cell membrane and organelles, with further cytotoxic effects on living cells [128]. Dietary supplementation with EOs is therefore a simple and convenient strategy to uniformly introduce natural antioxidants into phospholipid membranes, where they may effectively inhibit the oxidative reactions by preventing the formation of radicals, by scavenging them, or by promoting their decomposition at their localized sites and appears as a more effective way of retarding lipid oxidation of animal products compared to post mortem addition [129,130].

## 5. Conclusions

The increasing pressure on the livestock industry to stop the use of antibiotics as growth promoters has initiated new research to find safe and efficient alternatives. The wide range of bioactive compounds included in the essential oils could boost their use as multifunctional feed supplements for animals. EOs contain several components that have the potential to positively manipulate gut microbiota and rumen fermentation, inhibit pathogenic bacterial growth, prevent tissue oxidation and, as a result, improve growth performance and products’ quality of livestock. However, their effectiveness in animal production has not yet been proven to be consistent and conclusive and some issues need to be addressed before their commercial application. For example, an optimal dose of essential oils application, depending on their type, should be addressed, since their use at high concentrations could induce cytotoxic effects on living cells. Although EOs are generally recognized as safe (GRAS) for use in the food and feed industry and the accumulation of their components in the body is unlikely due to their rapid elimination (glucuronides by the kidneys) or exhalation (carbon dioxide), no analytical methods suitable for their identification and quantification (traceability, toxicity, possible residues, etc.) have yet been developed. At the same time, the beneficial effects of EOs dietary supplementation should be such as to justify the additional cost of their application. Finally, despite the fact that the understanding of EOs mode of action is a prerequisite for their regular application in animal production, our knowledge regarding their activities in animal (as well as in human) organism is still rather limited and there is a strong need for information regarding their absorption, distribution, metabolism and excretion. A further clarification of the above inquiries would therefore be beneficial for establishing EOs regular application in animal production.

## Figures and Tables

**Table 1:** Effects of essential oils or their components on gut microflora, morphology, enzyme activity and growth performance parameters in monogastric animals.

Essential Oil or Component	Level	Animal	Effects	Reference
Artemisinin	17 ppm	Broilers	Reduction of oocyst output and lesion scores attributable to *Eimeria tenella*.	[27]
BEO (thymol, eugenol and piperine)	100–200 mg/kg	Broilers	No effect on intestinal numbers of *C. perfringens*, GP and FCR. Reduction of FBW.	[28]
BEO (thymol, eugenol and piperine)	100 mg/kg	Broilers	No effect on FI, BWG, FCR, CT and ileal bacterial count (*C. perfringens* and Gram^−^ bacilli). Increase of ileum length and ileal villi height.	[29,30]
BEO (carvacrol, thymol, eucalyptol, lemon)	125–500 mg/kg	Broilers	Improvement of BWG and FCR (125 or 250 mg/kg). Reduction of *Salmonella* Heidelberg colonization in crops (500 mg/kg). No effect on *Salmonella* Heidelberg caecal or faecal counts.	[31]
BEO (cinnamaldehyde and thymol)	100 mg/kg	Broilers	No effect on ADG, FI, gut morphology and ileal bacterial count. Improvement of FCR and apparent ileal nitrogen digestibility.	[32]
BEO (cinnamaldehyde and thymol)	100 mg/kg	Broilers	No effect on FI. Improvement of BWG and FCR. Reduction of *Salmonella*-positive caecal samples.	[33]
BEO (garlic, sage, echinacea, thyme, oregano)	1 g/kg	Broilers	No effect on BWG, FI, FCR, CT and lesion score. Reduction of oocyst counts 6–14 days post infection.	[34]
BEO (oregano, laurel leaf and lavender)	50 mg/kg	Broilers	No effect on BWG, FI, FCR, intestinal length and caecal weight. Reduction of faecal *Eimeria* oocyst output.	[35]
BEO (oregano, cinnamaldehyde, carvacol, yucca extract)	250 mg/kg	Broilers	No effect on BWG and FI. Improvement of FCR, ATTD of DM and gross energy. Reduction of lesion score and *C. perfringens* and *E. coli* intestinal counts.	[36]
BEO (Agrimonia eupatoria, Echinacea angustifolia, Ribes nigrum and Cinchona succirubra extracts)	0.5–1.0 g/kg	Broilers	No effect on caecal lesion score. Improvement of BWG and FCR. Reduction of *Eimeria tenella* oocysts count and bloody diarrhea intensity.	[37]
BEO (oregano, anis and citrus peel)	125 mg/kg	Broilers	No effect on BWG, FI, intestinal pH values, caecal TVFA levels and total ileum microbiota counts. Improvement of FCR. Reduction of ileum ammonia concentration.	[38]
BEO (clove and cinnamon)	100 mg/kg	Broilers	No effect on FBW, ADG, FCR and CT.	[39]
BEO (capsaicin, cinnamaldehyde, carvacrol)	150–300 mg/kg	Broilers	No effect on FBW, ADG, FCR, CT and ileal ND. Reduction of rectal *E. coli* and *Clostridium perfringens* counts.	[40]
BEO (capsaicin, cinnamaldehyde, carvacrol)	100 mg/kg	Broilers	No effect on FBW, CT and ileal ND. Improvement of FCR. Increase of LAB counts and lipase activity in pancreas and intestine wall. Reduction of intestinal *E. coli* and *Clostridium perfringens* counts.	[22]
BEO (capsicum oleoresin, cinnamaldehyde, carvacrol)	100 mg/kg	Broilers	No effect on FBW. Improvement of FCR. Increase of mucus secretion intensity and accumulation inside cells of the gastrointestinal mucosa. Reduction of intestinal *E. coli* and *Clostridium perfringens* counts.	[19]
BEO (thymol, eugenol and piperine)	50 mg/kg	Broilers	No effect on FBW, ADG, FI, FCR and LAB counts. Increase of pancreatic trypsin, pancreatic alpha-amylase and intestinal maltase activity. Reduction of *E. coli* counts in ileo-caecal digesta.	[21]
BEO (basil, caraway, laurel, lemon, oregano, sage, tea and thyme)	30 mg/kg	Broilers	No effect on FI. Improvement of FBW, ADG, FCR and CT. Increase of caecal villus surface area.	[41]
BEO (carvacrol, 1,8-cineole, camphor, thymol, oregano EO, laurel leaf EO and lavender EO)	75 mg/kg	Broilers	No effect on CT. Negative effect on FCR. Reduction of FBW, ADG, FI, caecum weight, intestinal length and faecal *Eimeria* spp. oocyst excretion.	[42]
BEO1 (thymol, eugenol and piperine) or BEO2 (thymol, carvacrol, eugenol and piperine)	100 mg/kg	Broilers	Reduction of intestinal *Clostridium perfringens* counts.	[43]
BEO (thymol, eugenol and piperine)	100 mg/kg	Broilers	No effect on BWG, FI, FCR, lesion scores and *Eimeria* sp. oocyst counts. Modulation of intestinal microbial communities.	[44,45]
BEO (thymol, eugenol and piperine)	300 mg/kg	Broilers	No effect on BWG, FI, FCR and caecal microbial population. Slight modulation of intestinal microbial population.	[46]
BEO (thymol, cinnamaldehyde)	15 + 5 mg/kg	Broilers	No effect on FI, FCR and caecal bacterial (LAB, *E. coli*, *Clostridium perfringens*) counts. Increase of BWG.	[47]
Capsaicin	5–20 mg/kg	Broilers	Reduction of *Salmonella Typhimurium* counts.	[48]
Grape Seed Proanthocyanidin Extract	12 mg/kg	Broilers	Increase of BWG. Reduction of lesion scores. Restoration of the antioxidant/oxidant system balance after the parasite infection.	[49]
Mushroom (Lentinus edodes or Tremella fuciformis) or herb (Astragalus membranaceus Radix) polysaccharide extracts	2 g/kg	Broilers	No effect on BWG, FI, FCR. Increase of bifidobacteria and LAB counts and reduction of Bacteroides spp. and *E. coli* counts.	[50,51]
Mushroom (Lentinus edodes) extract	100 g/L water	Broilers	No effect on BWG, FI, FCR and CT. Promotion of bifidobacteria growth.	[52]
Oregano EO	300 mg/kg	Broilers	No effect on FBW and CT. Improvement of FCR. Reduction of FI and excreta oocyst counts.	[53]
Oregano EO	250–500 g/kg	Broilers	No effect on BWG, FI, FCR, digesta pH, weight and height of the intestinal parts and lipase and amylase activity. Increase of chymotrypsin activity and CPD.	[54]
Oregano EO	12–24 mg/kg	Broilers	No effect on BWG and FI. Improvement of FCR, intestinal morphological development and enzymatic activities (amylase).	[55]
Oregano EO	300 mg/kg	Broilers	No effect on FI. Improvement of BWG and FCR. Reduction of lesion score and *Eimeria tenella* oocyst counts.	[56]
Oregano EO	5–10 μL/kg	Broilers	Intense bacteridical action against lactobacilli and *E. coli* in faecal samples.	[57]
Oregano or garlic EO	300 mg/kg	Broilers	No effect on FI, FCR, CT, ileal Streptococcus, LAB and CB counts. Oregano EO results in reduced FBW (no effect of garlic EO). Reduction of ileal *Clostridium perfringens* counts.	[58]
Oregano EO	500 mg/kg	Broilers	Improvement of BWG and FCR. Reduction of coccidiosis lesion scores and faecal oocyst counts.	[59]
Oregano EO	600 mg/kg	Broilers	Improvement of BWG and FCR. No effect on LAB counts, but decrease of *E. coli* counts.	[60]
Oregano EO	0.5–1 g/kg	Broilers	No effect on intestinal villous height and crypt depth. Reduction of *Eimeria tenella* oocyst counts.	[61]
Oregano EO	300–600 mg/kg	Broilers	No effect on BWG and FI. Improvement of FCR. Reduction of lesion score and faecal *Eimeria* sp. oocyst counts.	[62]
Oregano EO	330 mg/kg	Broilers	No effect on FCR and lesion scores. Increase of FBW, FI and reduction of caecal *Clostridium perfringens* counts.	[63]
Peppermint EO	400 mg/kg	Broilers	No effect on BW, ADG, FI and FCR, faecal DMD and CPD and intestinal morphology.	[64]
Renga renga lily or Acacia extract	10 g/kg	Broilers	No effect on BWG, FI, FCR, intestinal morphology and ileal CB and *Clostridium perfringens* counts. Increase of ileal LAB counts.	[65]
Sophora flavescens extract	6–30 g/L water	Broilers	Increase of BWG. Reduction of lesion scores and faecal *Eimeria tenella* oocyst counts.	[66]
Thyme EO	1 g/kg	Broilers	No effect on FCR, AME, ATTD and intestinal microflora populations. Increase of BWG and FI.	[67]
Thyme EO	1 g/kg	Broilers	No effect on ADG, FI, FCR, DMD, CPD and TVFA. Reduction of caecal isobutyric+ isovaleric levels.	[68]
Thyme EO	0.5 g/kg	Broilers	No effect on caecal and large-intestinal bacterial counts. Improvement of intestinal barrier integrity and antioxidant status.	[69]
Thymol or cinnamaldehyde or BEO (thymol, eugenol and piperine)	100 mg/kg	Broilers	No effect on FBW, ADG, FI, FCR, ileal CPD and activity of digestive enzymes in intestinal contents and pancreatic tissue.	[70]
Tulbaghia violacea extract	35 mg/kg	Broilers	No effect on BWG. Improvement of FCR and reduction of *Eimeria* sp. oocyst counts.	[71]
Thyme EO	60 mg/kg	Quails	No effect on FI, intestinal parameters and CT. Improvement of BWG and FCR. Reduction of abdominal fat levels.	[72]
Thyme EO	1 g/kg	Quails	No effect on FI and FCR. Increase of FBW, carcass weight ileal LAB counts and reduction of ileal *E. coli* counts.	[73]
Artemisia annua L. extract	2.5–5.0 mL/kg	Rabbits	Reduction of faecal oocytes and caecal TBC. Improvement of FCR and RGR.	[74]
Thyme EO	0.5 g/kg	Rabbits	No effect on faecal and caecal bacterial counts. Improvement of intestinal integrity and antioxidant status.	[75]
BEO (thymol, eugenol, piperine)	30 mg/kg	Turkeys	Improvement BWG, FCR and antioxidant status. Increase of caecal LAB counts and reduction of caecal CB counts.	[76]
BEO (thyme, rosemary, oregano)	100 mg/kg	Grower-finisher pigs	Improvement of ADG, FCR, GED, CPD (grower period) and ADG (grower-finisher period). Reduction of ammonia excretion.	[77]
BEO (buckwheat, thyme, curcuma, black pepper and ginger)	250–500 mg/kg	Grower pigs	No effect on FCR. Increase of BWG, FI and reduction of faecal noxious gas (ammonia and hydrogen sulfide) content.	[78]
BEO (oregano, anise, orange peel, and chicory EOs)	125 mg/kg	Weaner pigs	No effect on ADG, FI, FCR. Improvement of DM and N digestibility. Reduction of faecal *Salmonella typhimurium* and *E. coli* counts. Increase of faecal *Lactobacillus* spp. count.	[79]
BEO (thymol and cinnamaldehyde)	0.1–0.15 mg/kg	Weaner pigs	Improvement of ADG and FCR. Increase of FI and faecal LAB counts. Reduction of diarrhea occurrence and faecal *E. coli* counts.	[80]
BEO (thymol and cinnamaldehyde)	100 mg/kg	Weaner pigs	Improvement of ADG, DMD, CPD and faecal score. Increase of villus height to crypt depth ratio in the jejunum, caecal LAB counts and reduction of caecal and rectal *E. coli* counts.	[81]
BEO (peppermint, anise and clove)	300 mg/kg	Weaner pigs	No effect on BWG, FI and gastrointestinal microbiota. Improvement of FCR, CPD and amino acids digestibility.	[82]
BEO (oregano, cinnamon and Mexican pepper)	150–300 mg/kg	Weaner pigs	No effect on BWG, FI, FCR and ATTD. Increase of gastric retention time and pH, lactobacilli:enterobacteria ratio and decrease of ileum total microbial count.	[83]
BEO (oregano, cinnamon and Mexican pepper)	300 mg/kg	Weaner pigs	No effect on BWG, FI, FCR, ATTD, intestinal pH and gastrointestinal morphology.	[84]
BEO (oregano, cinnamon and Mexican pepper)	200 mg/kg	Weaner pigs	No effect on BWG, FI and FCR. Increase of ileal lactobacilli:enterobacteria ratio and decrease of TVFA production in the cecum.	[85]
BEO (carvacrol and thymol)	500–1500 mg/kg	Weaner pigs	No effect on BWG, FI, FCR, praecaecal digestibility, enzyme activities, faecal microbial counts and intestinal microflora.	[86,87]
BEO (cinnamon, thyme and oregano extract)	750 mg/kg	Weaner pigs	No effect on BWG, FI, FCR, intestinal morphology and LAB counts. Reduction of CB counts.	[88]
BEO (buckwheat, thyme, curcuma, black pepper and ginger)	250 mg/kg	Weaner pigs	No effect on ADG, FI, FCR and caecal LAB counts. Improvement of CPD and DMD and reduction of faecal *E. coli* counts.	[89]
BEO (thymol, cinnamaldehyde)	250 mg/kg	Weaner pigs	No effect on FI, caecal *E. coli* and LAB counts. Improvement of ADG, FCR, DMD, CPD and GED. Increase of villus height (jejunum) and reduction of rectal *E. coli* counts.	[90]
Phytoncide	2 g/kg	Weaner pigs	No effect on ADG, FI, FCR, faecal *E. coli* counts and diarrhea scores. Improvement of DMD and increase of faecal LAB counts.	[91]
Powder of medicinal herbs (Panax ginseng, Dioscoreaceae opposite, Atractylodes macrocephala, Glycyrrhiza uralensis, Ziziphus jujube and Platycodon grandiflorum)	1–3 g/kg	Weaner pigs	No effect on FI, BWG and FCR. Increase of DMD, CPD, GED, villous height and ileal and caecal LAB counts and reduction of caecal CB counts.	[92]

ADG: average daily gain; AME: apparent metabolisable energy; ATTD: apparent total tract digestibility; BEO: blend of essential oils; BWG: body weight gain; CB: coliform bacteria; CT: carcass traits; CPD: crude protein digestibility; DM: dry matter; DMD: dry matter digestibility; DT: digestive tract; EO: essential oil; FBW: final body weight; FCR: feed conversion ratio; FI: feed intake; ND: nutrients digestibility; GED: gross energy digestibility; GP: growth performance; LAB: lactic acid bacteria; RGR: relative growth rate; TBC: total bacterial counts; TVFA: total volatile fatty acids.

**Table 2:** Effects of essential oils or their components on rumen characteristics and performance parameters.

Essential Oil or Component	Level	Animal	Effects	Reference
Eugenol	50 mg/kg DM	Dairy cows	No effect on DMI, ND, RP and MY.	[100]
Cinnamaldehyde and eugenol	85 & 140 mg/day, respectively	Dairy cows	No effect on DMI, FB, VFA concentration, NH_3_, A:P ratio, ruminal pH, ND and MY.	[101]
Cinnamaldehyde and eugenol	1.7 & 2.8 g/day, respectively	Dairy cows	No effect on DMI, FB, VFA concentration, NH_3_, A:P ratio, ruminal pH, ND and MY.	[101]
Garlic EO	5 g/day	Dairy cows	No effect on DMI, ruminal pH, VFA concentration, NH_3_ and MY. Improvement of FD in rumen.	[102]
Juniper berry EO	2 g/day	Dairy cows	No effect on DMI, ruminal pH, VFA concentration, NH_3_ and MY. Improvement of FD in rumen.	[102]
Oregano leaves	250, 500 or 750 g/day	Dairy cows	No effect on ND, VFA concentration, ruminal pH and MY. Dose-dependent decrease of DMI and MP.	[103]
Oregano leaves	500 g/day	Dairy cows	No effect on DMI, ND, VFA concentration, ruminal pH and MY. Decrease of MP.	[104]
MEO	2 g/day	Dairy cows	No effect on FI, ND, VFA concentration and MY. Increase of ruminal pH.	[105]
MEO	750 mg/day	Dairy cows	No effect on FI, ND, RP and MY. Increase of ruminal pH.	[106]
MEO	1.2 g/day	Dairy cows	No effect on BW, BCS and VFA concentration. Increase of DMI and MY.	[107]
Blend of oregano, cinnamon, thyme and orange peel EOs	0.32, 0.64 or 0.96 g/day	Dairy cows	No effect on DMI, ND and MY.	[108]
Mixture of eugenol, geranyl acetate and coriander oil	1 g/day	Dairy cows	No effect on DMI, ND and MY. Decrease of BCS.	[109]
MEO	1 g/day	Steers	No effect on DMI, ADG, CC, ND, VFA concentration and ruminal pH.	[110]
Cinnamon EO	5 g/day	Calves	No effect on DMI, ADG, VFA concentration and ruminal pH. Decrease of A:P ratio.	[111]
Thyme EO	5 g/day	Calves	No effect on DMI, ADG, VFA concentration and ruminal pH. Decrease of A:P ratio.	[111]
Anise extract	500 mg/day	Beef heifers	Reduction of VFA concentration and RP (A:P ratio, NH_3_).	[112]
Cashew and castor EOs	3 g/day	Bulls	No effect on DMI, ND and CC. Improved ADG, final BW and FE.	[113]
Coconut oil	7%	Swamp buffaloes	Negative effect on DMI, VFA concentration, A:P ratio and MP. No effect on ruminal pH, NH_3_ and ND.	[114]
Eucalyptus EO	2 mL/day	Swamp buffaloes	No effect on DMI, ND, ruminal pH, VFA concentration. Decrease of MP.	[115]
Carvacrol	200 mg/kg DM	Lambs	Reduction of ruminal pH and increase of VFA concentration. No effect on other RP (A:P ratio, NH_3_), DMI, AVG, CC and MC.	[116]
Cinnamaldehyde	200 mg/kg DM	Lambs	Reduction of ruminal pH, increase of VFA concentration. No effect on other RP (A:P ratio, NH_3_) DMI, AVG, CC and MC.	[116]
Cinnamaldehyde	200 mg/kg DM	Lambs	No effect on RP, DMI, CC and MC. Positive effect on AVG.	[117]
Coconut oil	25, 50 or 75 g/kg of CF	Lambs	No effect on ADG and CC. Decrease of DMI.	[118]
Garlic EO	200 mg/kg DM	Lambs	No effect on RP, DMI, AVG, CC and MC.	[117]
Juniper berry EO	200 mg/kg DM	Lambs	No effect on RP, DMI, CC and MC. Positive effect on AVG.	[117]
MEO	50, 100 or 150 mg/kg DM	Dairy ewes	No effect on DMI and ruminal pH. Positive dose-dependent effect on MY.	[119]
Garlic EO	5 g/kg DM	Sheep	No effect on DMI and MP.	[120]
Mixture of clove, oregano, cinnamon, and lemon EOs	1 g/day	Sheep	No effect on DMI, ND and ruminal pH. Decrease of VFA concentration, A:P ratio and RPD.	[121]
Mixture of eugenol, carvacrol, citral and cinnamaldehyde	1 g/day	Sheep	No effect on DMI, ND and ruminal pH. Decrease of VFA concentration, A:P ratio and RPD.	[121]
Resveratrol	0.25 g/day	Sheep	No effect on DMI, ruminal pH, NH_3_ and VFA concentration. Positive effect on ND. Decrease of MP and A:P ratio.	[122]
Mixture of linalool, *p*-cymene, *alpha*-pinene & *beta*-pinene	0.043 or 0.43 g/kg DM	Dairy goats	No effect on DMI, ND, VFA concentration, A:P ratio and MY.	[123]

ADG: average daily gain, A:P ratio: acetate: propionate ratio, BCS: body condition score, BW: body weight, CC: carcass characteristics, CF: concentrated feed, DM: dry matter, DMI: dry matter intake, FB: feeding behavior, FD: feed digestibility, FE: feed efficiency, FI: feed intake, MC: meat characteristics, MEO: EOs components mixture (thymol, eugenol, vanillin, guaiacol and limonene), MP: methane production, MY: milk yield, ND: nutrients digestibility, NH_3_: ammonia, RP: rumen parameters, RPD: ruminal protein digestibility, VFA: volatile fatty acids.

**Table 3:** Dietary supplementation of animal diets with the two most common essential oils derived from the Greek flora (oregano and rosemary) and effects on the quality of the derived products.

Essential Oil	Major Antioxidant Compounds	Level	Product	Effect	Reference
Oregano	*p*-cymene, gamma-terpinene, beta-Caryophyllene, borneol, carvacrol, thymol	100–200 mg/kg	Egg	+	[131]
		100 mg/kg	Chicken Meat	+	[132]
		50–100 mg/kg	Chicken Meat	+	[133,134,135]
		300 mg/kg	Chicken Meat	NS	[136]
		500 mg/kg	Chicken Meat	+	[137]
		1.0 mL/kg	Lamb Meat	+	[138]
		0.25–1.0 mL/kg	Pork Meat	NS	[139]
		500 mg/kg	Pork Meat	NS	[140]
		100–200 mg/kg	Rabbit Meat	+	[141]
		100 mg/kg	Turkey Meat	+	[14,142]
		200 mg/kg	Turkey Meat	+	[129,143]
		1 mL/kg	Sheep Milk	NS	[144]
Rosemary	Carnosic acid, carnosol, rosmanol, rosmarinic acid	200 mg/kg	Quail egg	+	[145]
		1.0 g/kg	Beef	+	[146]
		400 mg/kg	Lamb Meat	NS	[147]
		0.6 g/kg	Lamb Meat	NS	[148,149]
		0.4 g/kg	Lamb Meat	+	[150,151]
		500 mg/kg	Chicken Meat	+	[152]
		100–200 mg/kg	Chicken Meat	+	[153]
		40 mg/kg	Pork Meat	+	[154]
		0.6–1.2 g/day	Sheep Milk	+	[155]

+: positive effect, NS: no effect.
